# Miro1 Enhances Mitochondria Transfer from Multipotent Mesenchymal Stem Cells (MMSC) to Neural Cells and Improves the Efficacy of Cell Recovery

**DOI:** 10.3390/molecules23030687

**Published:** 2018-03-19

**Authors:** Valentina A. Babenko, Denis N. Silachev, Vasily A. Popkov, Ljubava D. Zorova, Irina B. Pevzner, Egor Y. Plotnikov, Gennady T. Sukhikh, Dmitry B. Zorov

**Affiliations:** 1 A. N. Belozersky Institute of Physico-Chemical Biology, Lomonosov Moscow State University, 119991 Moscow, Russia; nucleus-90@yandex.ru (V.A.B.); silachevdn@genebee.msu.ru (D.N.S.); popkov.vas@gmail.com (V.A.P.); lju_2003@list.ru (L.D.Z.); irinapevzner@mail.ru (I.B.P.); 2 V. I. Kulakov National Medical Research Center of Obstetrics, Gynecology and Perinatology, 117997 Moscow, Russia; gtsukhikh@mail.ru; 3 Institute of Molecular Medicine, Sechenov First Moscow State Medical University, 119991 Moscow, Russia; 4 Department of obstetrics, gynecology, perinatology and reproduction, Sechenov First Moscow State Medical University, 119991 Moscow, Russia

**Keywords:** ischemia, tunneling nanotubes, stroke, mitochondria, astrocyte

## Abstract

A recently discovered key role of reactive oxygen species (ROS) in mitochondrial traffic has opened a wide alley for studying the interactions between cells, including stem cells. Since its discovery in 2006, intercellular mitochondria transport has been intensively studied in different cellular models as a basis for cell therapy, since the potential of replacing malfunctioning organelles appears to be very promising. In this study, we explored the transfer of mitochondria from multipotent mesenchymal stem cells (MMSC) to neural cells and analyzed its efficacy under normal conditions and upon induction of mitochondrial damage. We found that mitochondria were transferred from the MMSC to astrocytes in a more efficient manner when the astrocytes were exposed to ischemic damage associated with elevated ROS levels. Such transport of mitochondria restored the bioenergetics of the recipient cells and stimulated their proliferation. The introduction of MMSC with overexpressed Miro1 in animals that had undergone an experimental stroke led to significantly improved recovery of neurological functions. Our data suggest that mitochondrial impairment in differentiated cells can be compensated by receiving healthy mitochondria from MMSC. We demonstrate a key role of Miro1, which promotes the mitochondrial transfer from MMSC and suggest that the genetic modification of stem cells can improve the therapies for the injured brain.

## 1. Introduction

The role of reactive oxygen species (ROS) in vital cellular functions has become an axiom; their role in signal transduction is important for cell growth, differentiation, and death [1]. Among these multi-faceted functions, special attention is deserved by recently discovered functions of ROS in mitochondrial traffic [2,3], which are directly related to the switch from normal to pathological cell functioning and vice versa. One option to provide normalization of a damaged tissue is to use cell technologies with stem cells of different varieties. Besides cell technologies, we currently observe a trend of developing mitochondrial technologies [4], which may significantly contribute to the therapy of damaged tissues. It has occurred that cell and mitochondrial technologies are inter-related, intercellular communication above all accounting for the interaction of stem cells with other cells. This interaction has increasingly become a target for researchers because of the great therapeutic potential of cell technologies. In general, cell–cell interactions occur in different ways. A stem cell can interact with the neighboring cells through paracrine factors that they produce [5], through microvesicles and endosomes that they release [6], through direct contact of cell surface receptors with intercellular cytoplasmic bridges (so-called tunneling nanotubes, TNT [7]), and lastly through fusion with other cells. The last two types of cell interaction can lead to the exchange of cellular contents including organelles, such as mitochondria [8].

Since 2004, when the intercellular transport of mitochondria was discovered [9], numerous studies have been focused on this process both as a fundamental phenomenon of different cell type interactions and, in a potentially practical way, as a basis for cell therapy. Since a large number of pathologies are associated with impaired mitochondrial functions [10,11,12], the possibility of replacing malfunctioning organelles by healthy donor mitochondria appears to be very promising. Many researchers have indicated that most often mitochondrial transfer is observed when one of the interacting cells is a stem cell [13,14]. A number of examples of mitochondria transfer was shown to occur through the formation of tunneling nanotubes, TNT, connecting multipotent mesenchymal stem/stromal cells (MMSC) with target cells of various tissues, leading to the transfer of the MMSCs’ mitochondria to cardiomyocytes, vascular smooth muscle cells, endothelial cells, pulmonary alveolar epithelial cells, renal tubular cells, and cancer cells [15,16,17,18,19]. These various studies clearly demonstrated that MMSC mitochondria could afford new properties to the recipient cells. Moreover, a protocol of direct and quantitative transfer of mitochondria has been designed recently, when mitochondria isolated from donor cells were introduced into recipient cells [20]. Caicedo and colleagues showed that the transfer of even a small number of MMSC-derived mitochondria resulted in enhanced oxidative phosphorylation and promotion of cell proliferation in recipient cells.

A detailed study of the mitochondrial traffic, both intracellular and intercellular, revealed that one of the key participants in this process is Miro1 (mitochondrial Rho-GTPase 1, synonym: RhoT1), a calcium-sensitive adaptor protein which connects the mitochondrion and the KIF5 motor protein through a set of accessory proteins like Miro2, TRAK1, TRAK2, and Myo19, thus organizing mitochondrial movement along microtubules inside the cells [21,22,23]. Miro1 plays a key role in the axonal transport of mitochondria in neurons, and alteration of Miro1 function was found to play a role in the incidence of pathologies such as Parkinson’s disease [24,25], amyotrophic lateral sclerosis [26], and schizophrenia [27]. The discovery of Miro1 as a principle element in the intercellular transport of mitochondria gave rise to speculations about the possibility of targeted modifications of stem cells in order to enhance their donation of mitochondria and therefore to increase their therapeutic efficacy. Recently, there have been few studies showing that a higher efficiency of the mitochondria transfer after overexpression of Miro1 was associated with enhanced positive effects of MMSC [28].

Given the importance of mitochondria for neuronal function and the association of mitochondrial dysfunction with a large number of neurodegenerative disorders and acute brain injury, unraveling the role of Miro1 in mitochondrial transfer in neural cells is highly important for human health and disease. In this study, we provide a comprehensive analysis of the transfer of mitochondria from MMSC to astrocytes and neuron-like PC12 pheochromocytoma cells. We evaluated the efficiency of the intercellular transfer of mitochondria under normal conditions, upon induction of mitochondrial damage in astrocytes and PC12, in part caused by oxidative stress associated with elevated ROS levels in the system, and after lentivirus-driven increased expression of Miro1 in MMSC. To assess the therapeutic potential of mitochondria transfer, we inspected the benefits of cell therapy in the treatment of experimental ischemic stroke in rats with MMSC overexpressing Miro1 protein.

## 2. Results

### 2.1. Transfer of Mitochondria from MMSC to Neural Cells

**Figure 1:**
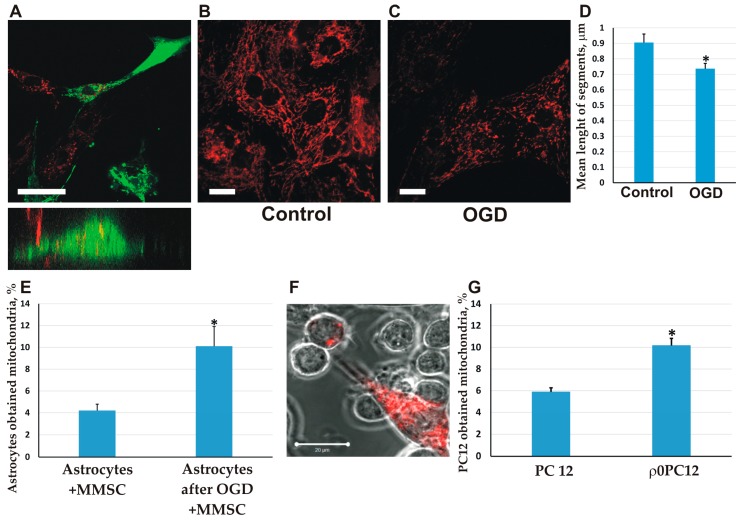
The transfer of mitochondria from MMSC to astrocytes and neural cells following mitochondrial damage. (**A**) Mitochondrial transfer from MMSCs to astrocytes. DsRed-labeled mitochondria from MMSC were transferred to astrocytes with GFP-labeled mitochondria. The intracellular location of red fluorescence was confirmed using confocal line-scanning microscope analysis of cells along the *z*-axis (bottom); (**B**–**D**) Mitochondrial fragmentation in astrocytes stained with TMRE (tetramethylrhodamine, ethyl ester) after 5 h of oxygen-glucose deprivation (OGD). Averagely shorter mitochondrial fragments (**D**) supports mitochondrial fragmentation; (**E**) The efficacy of mitochondrial transfer from MMSС to astrocytes is increased after OGD; (**F**) The transfer of DsRed-labeled mitochondria from ММСК to *ρ0* PC12 cells; (**G**) MMSCs more efficiently transferred mitochondria to *ρ0* PC12 cells than to native PC12 cells. Scale bars = 10 µm (**A**, **B**), and 20 µm (**F**). All experiments were performed at least in triplicate; * denotes significant differences between groups (*p* < 0.05) (One-way ANOVA, followed by Tukey’s post hoc analysis). Values are given as mean ± standard error of the mean (SEM).

Since we have previously observed the directed transfer of mitochondria from MMSC to neurons during their co-cultivation [29], in this paper we explored mitochondrial transport from MMSC into astrocytes. To visualize mitochondria, we transfected MMSC with lentiviral constructs encoding red fluorescent protein fused with a mitochondrial localization signal (mitoDsRed). Astrocytes were transfected with a similar construct, encoding green fluorescent protein with a mitochondrial localization signal (mitoGFP). After 2 days of co-cultivation of these two types of cells, we observed red-fluorescing mitochondria within astrocytes among their own green fluorescing mitochondria (Figure 1A). Notably, in the MMSC after co-cultivation, we were not able to detect green-fluorescing mitochondria coming from astrocytes. The confirmation of the intracellular location of the transferred mitochondria was obtained using a confocal line-scanning microscope to analyze cells along the *z*-axis, ultimately demonstrating that the red-fluorescing mitochondria in the astrocyte were surrounded by their own green mitochondria (Figure 1A).

Further, we analyzed how cellular damage caused by ischemia/reoxygenation of astrocytes affected the transfer of mitochondria from MMSC. A conventional cellular model of brain ischemia in vitro is the oxygen-glucose deprivation (OGD), highly associated with oxidative stress caused by elevated production of ROS [30,31], which was applied to the astrocyte culture for 5 h. As a result of OGD, the mitochondria within these cells became remarkably fragmented (Figure 1B–D), indicating their damage [32]. We found that in the culture of astrocytes exposed to OGD for 5 h and further co-cultivated with MMSC, the fraction of astrocytes that received mitochondria from the stem cells was significantly increased (almost doubled) (Figure 1E). This means that mitochondrial damage in targeted cells (astrocytes) stimulated the transport of functional mitochondria from MMSC to astrocytes.

The activation of mitochondrial transfer to the recipient cells with damaged mitochondria was also demonstrated in neuron-like PC12 cells. The PC12 cell line was cultured in the presence of ethidium bromide for three weeks, which resulted in cells either containing broken mitochondrial DNA or completely lacking it (*ρ0* cells). Ultimately, these cells were not capable of oxidative phosphorylation and the synthesis of uridine [33]. Co-cultivation of such cells with MMSC also caused a significant rise in the fraction of PC12 cells that received mitochondria from MMSC (Figure 1F,G).

### 2.2. The Transfer of Mitochondria Can Occur through Tunneling Nanotubes

**Figure 2:**
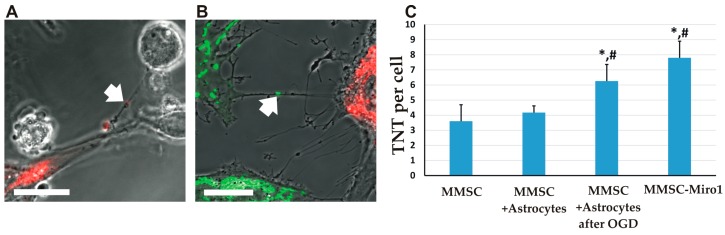
Mitochondria transfer from MMSCs to neural cells is supported by tunneling nanotubes (TNT). Formation of TNT between MMSC with DsRed-labelled mitochondria and unlabeled PC12 cells (**A**) and MMSC with GFP-labelled mitochondria and DsRed-labelled astrocytes (**B**); MMSC-derived mitochondria are seen in TNT (arrows). More TNTs are observed after OGD or overexpression of Miro1 in MMSC (**C**). Scale bars = 20 µm (**A**,**B**). All experiments were performed at least in triplicate; *,# denotes significant differences with respect to the MMSC group (*p* < 0.05) or the MMSC + Astrocytes group, (One-way ANOVA, followed by Tukey’s post hoc). Values are given as mean ± standard error of the mean (SEM).

It is important to note that in co-cultures of MMSC with either astrocytes or PC12, the formation of TNT was observed (Figure 2), which, according to previous data, could provide transfer of mitochondria [9,19]. The average number of TNT found in MMSC increased when they were co-cultivated with astrocytes, compared with MMSC monoculture (Figure 2C). When MMSC were co-cultivated with astrocytes subjected to OGD, the number of TNT was increased even more (Figure 2C). A similar rise in TNT formation was observed for MMSC overexpressing Miro1 after they were co-cultivated with astrocytes (Figure 2C).

### 2.3. The Transport of Mitochondria Restores Cell Proliferation and Respiration

**Figure 3:**
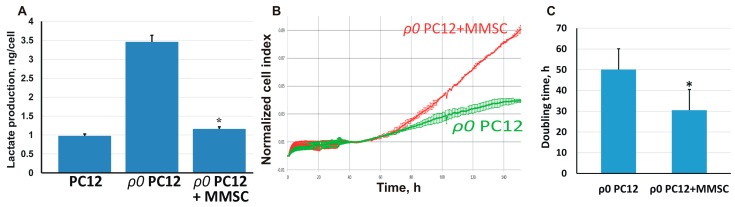
Retardation of glycolysis and higher proliferation in *ρ0* PC12 cells associated with the transfer of mitochondria from MMSC. (**A**) Co-cultivation of MMSC and *ρ0* PC12 cells was associated with lower production of lactate, possibly speeding ATP production from oxidative phosphorylation and blocking glycolysis, thus increasing the normalized cell index (**B**) and reducing the doubling time (**C**), which demonstrated the activation of proliferation in PC12 cells. All experiments were performed at least in triplicate; * denotes significant differences between groups (*p* < 0.05) (One-way ANOVA, followed by Tukey’s post hoc analysis). Values are given as mean ± standard error of the mean (SEM).

An important functional result of the mitochondria transfer from MMSC was the restoration of cell functions in the recipient cells. Thus, *ρ0* PC12 cells with damaged mitochondrial DNA produced the main part of their energy by anaerobic glycolysis because of the disrupted respiration, yielding a significant increase in lactate concentration in the culture medium (Figure 3A). After co-cultivation of such cells with MMSC, the concentration of lactate in the medium almost reached control values, indicating a recovery of oxidative phosphorylation and switching of *ρ0* PC12 cells to aerobic respiration instead of glycolysis.

In addition, the proliferation rate of *ρ0* PC12 cells was significantly less than that of the controls. After co-cultivation with MMSC, we separated PC12 cells using magnetic sorting, analyzed their growth rate (Figure 3B), and found that the proliferation of *ρ0* PC12 cells after co-cultivation was significantly higher, with the doubling time of the population decreasing almost twofold (Figure 3C). Thus, co-cultivation with MMSC not only restored the bioenergetics of the PC12 cells but also normalized their proliferation, which had been impaired by mitochondrial damage.

### 2.4. Effect of Miro1 Overexpression on Mitochondrial Transfer

**Figure 4:**
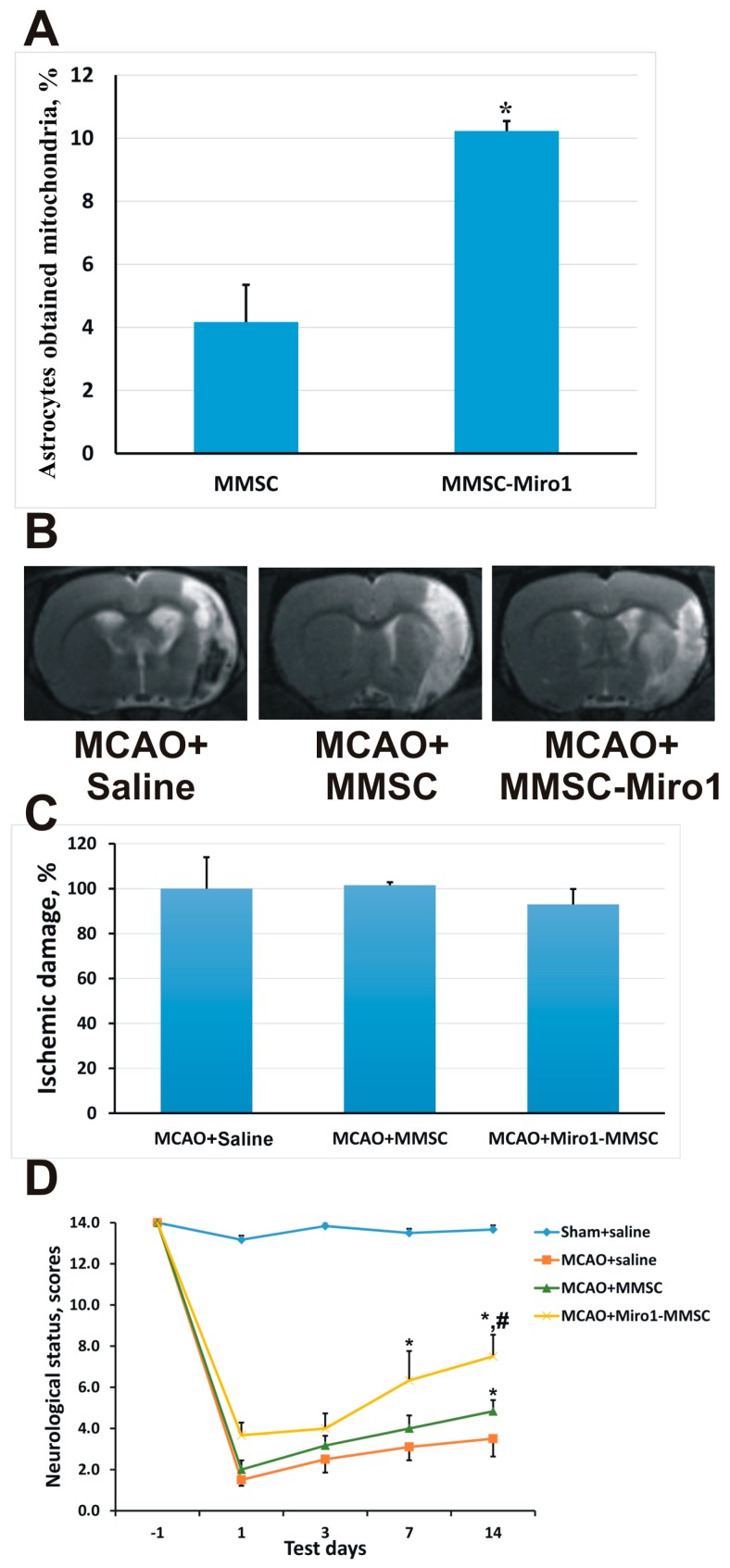
Beneficial effects of Miro1 overexpression on mitochondrial transfer. (**A**) Efficacy of mitochondrial transfer from MMSC overexpressing Miro1 to astrocytes after 24 h of co-cultivation; (**B**) Representative T2-weighted MR-images from coronal brain sections obtained 14 days after middle cerebral artery occlusion (MCAO). Hyperintensive regions refer to ischemic areas; (**C**) The volume of the ischemic lesions in the brain on day 14 after MCAO; (**D**) Effect of MMSC transplantation on the neurological status at different times after stroke. Intravenous injection with either native MMSCs or MMSC-Miro1 caused a significant decrease of the neurological deficit, with MMSC-Miro1 being more effective. *, *p* < 0.05 vs. ischemic saline controls; #, *p* < 0.05 vs. ischemic MMSC-treated rats. All cell culture experiments were performed at least in triplicate, and at least six animals were used for each group in the stroke study.

We previously described that MMSC showed increased levels of Miro1 after co-cultivation with neurons [29]. It was recently discovered that Miro1 (apart from its role in intracellular transport) is also responsible for the intercellular transfer of mitochondria [34]. We assessed Miro1 potential to enhance the MMSC ability to donate mitochondria to neural cells. To achieve this, we created a lentiviral construct containing the Miro1 gene, which, when expressed in MMSC, increased the intracellular levels of Miro1 in these cells. When the Miro1-MMSC were co-cultivated with astrocytes, the latter received more mitochondria from the MMSC (Figure 4A).

The most important result of the transfer of mitochondria from the MMSC was the restoration of neural functions observed in vivo. We simulated ischemic stroke in rats, resulting in a neurological deficit which was observed as a dysfunction of the limb innervated from the damaged regions of the brain cortex (Figure 4C,D). After intravenous injection of MMSC, the neurological deficit was reduced after 14 days of observation, but the most pronounced therapeutic effect was observed when MMSC with overexpressed Miro1 were administered to rats (Figure 4D). Thus, we can conclude that the transfer of mitochondria is a very important component of the neuroprotective effect of MMSC after experimental ischemic stroke.

## 3. Discussion

It is well known that ischemic stroke is associated with oxidative stress and severe impairments of mitochondrial functioning in the damaged brain region [35]. Numerous studies have demonstrated that the protection of the mitochondria of neural cells from damage can be an effective therapeutic approach for the treatment of acute neurological disorders [36,37,38,39]. Most of these strategies consisted in either antioxidant usage to decrease excessive levels of ROS or in the induction of signaling cascades associated with preconditioning that ultimately prevented the increase of mitochondrial permeability. However, the regulation of mitochondrial transfer or the donation of mitochondria from stem cells may also be a very promising strategy to protect cells of the nervous tissue from the deleterious effects of ischemia.

Intercellular mitochondrial transport was discovered in 2006 [19], and, in the following decade, this process was shown to be associated with the rescue of aerobic respiration and the restoration of mitochondria functioning in the recipient cells [17,19]. In this study, we demonstrated that mitochondrial transfer into a defective recipient neural cell in vitro was associated with the restoration of respiration and proliferation of the cells harboring damaged mitochondria. In addition, we presented the benefits of a mitochondrial donation from mesenchymal stem cells in vivo in models of ischemic stroke. We also elucidated the role of Miro1, which promotes mitochondrial transfer from MMSC and showed that stem cells can be genetically improved to create more effective therapies for damaged brain areas.

The mechanisms of mitochondrial donation by stem cells have been recently investigated in numerous models, highlighting the role of TNT as directional devices and of microvesicles as vehicles for mitochondrial transfer in vitro [15] and in vivo [17,40]. In our previous work, we found that mitochondrial transport was part of a mechanism of general exchange of cellular content. We have already demonstrated that MMSC are able to communicate with different types of differentiated cells through TNT and can exchange cytoplasm and mitochondria. However, while we showed that the transfer of cytoplasm could occur in both directions (from MMSC and toward MMSC), depending on the type of recipient cells, mitochondria were always transferred unidirectionally: from MMCS to differentiated cells [18,29,41].

Ahmad and colleagues recently showed that the traffic of mitochondria from stem cells to recipient cells is regulated by Miro1 and is part of a well-tuned specialized process, rather than a general diffusion-operated exchange with cellular contents [28]. It is believed that the movement of mitochondria through cytoplasmic bridges such as TNT occurs via microtubules containing Miro1 cargo [34]. Recent findings showed that ROS control, particularly, mitochondrial motility mediated by Miro1 [3], demonstrating the role of ROS and mitochondrial traffic in the transition from normal to abnormal cells and vice versa.

In this study, we confirmed the importance of Miro1 for the transport of mitochondria in neural cells and its contribution to the therapeutic efficacy of MMSC following an experimental stroke. Apart from the direct enhancement of in vitro mitochondrial transfer efficiency, we found that overexpression of Miro1 increased the potency of MMSC to alleviate neurological deficit after stroke.

We also showed that the efficiency of mitochondria transfer from MMSC was increased when the recipient cell harbored damaged mitochondria. We provoked mitochondrial injury in astrocytes by inducing ischemia in vitro (oxygen-glucose deprivation known to be associated with elevated ROS production) [30,31], and in PC12 by long-term incubation with ethidium bromide which causes mitochondrial DNA damage. In both models, after co-culturing with MMSC, we observed increased transport of MMSC-derived mitochondria, which was also associated with increased formation of TNT. This may be considered as another proof of concept that the transport of mitochondria occurs, to a large extent, via TNT, and the observation of mitochondria-targeted fluorescent proteins inside these nanotubes gave a significant support to this conclusion.

Interestingly, after MMSC were co-cultured with *ρ0* PC12 cells that had defective or deleted mtDNA, some of the *ρ0* PC12 cells acquired functional mitochondria. The rescued cells were able to proliferate exponentially in a standard medium without the addition of uridine and pyruvate, similar to the parental PC12 cells. Although our results do not fully exclude the possibility that the cells underwent fusion, this is unlikely because we never detected two nuclei in cells with transferred mitochondria. The restoration of mitochondrial function was demonstrated by the accelerated growth and higher oxidative bioenergetics expressed as a decreased production of lactate.

It is believed that numerous neurological diseases are associated with mitochondrial dysfunction as a result of genetic disorders in mitochondrial DNA or nuclear genes encoding mitochondrial proteins [42], and as a result of mitochondrial damage in acute pathologies, e.g., ischemia [10]. Our data suggest that the consequences of such mitochondrial defects could be compensated by the transfer of healthy mitochondria from MMSC. Although, according to our data, such transport is not a very frequent phenomenon, it can be observed not only in vitro but also in vivo [17,28,29]. Even without MMSC, the transfer of healthy mitochondria from other cells may rescue damaged neurons. Thus, transient focal cerebral ischemia in mice induced the entry of astrocytic mitochondria into adjacent neurons, and this entry amplified cell survival signals. Suppression of CD38 signaling by short interfering RNA molecules reduced extracellular mitochondria transfer and worsened neurological outcomes [43]. The donation of mitochondria could partially explain the positive outcomes observed following the transplantation of MMSC in experimental models of stroke [29], spinal cord injury [44], or neurodegenerative pathologies [45], because the long-term engrafting of allogenic MMSC in tissues was rarely observed and it is unlikely to mediate the therapeutic effect of MMSC transplantation.

Of course, the positive effect arousing from stem cells may involve other mechanisms besides the transport of healthy mitochondria from the stem cells to injured cells. It has been repeatedly shown that the positive effect of a stem cell located near an affected cell can probably be attributed to paracrine and endocrine activities, which were evaluated in studies of the stem cell secretome [46,47]. In a recent paper [48], all paracrine factors released by stem cells contributing to the repair of damaged heart tissue were reviewed in detail. Similar examples can be given for lymphoid stem cells (T cells and B cells) that release paracrine factors that can heal affected skeletal muscles [49] and other stem cells releasing either neurotropic factors in response to brain cell damage [50,51] or factors contributing to the restoration of a damaged kidney [52]. However, mitochondria transfer from stem cells as a response to SOS signal(s) sent by damaged cells into to the environment is another dimension of a complex interplay in cell interactions where the stem cell is a key player providing aid to injured cells or tissues.

## 4. Materials and Methods

### 4.1. Preparation of Astroglial Cells

Astroglial cultures were prepared from the cerebral cortical tissue of 1–2-day-old outbred white rats according to [53]. After removal of the meninges, the cerebral cortices were dissected, and the remaining brain tissue was incubated for 30 min in trypsin/EDTA (0.05/0.02% wt/vol in PBS) at 37 °C; the cortex tissue pieces were rinsed with PBS and complete media (DMEM/F12 supplemented with 10% fetal bovine serum (FBS) and 0.5 mM l-glutamine) and then dissociated by pipetting, and the cell suspension was applied to poly-l-lysine-coated flasks. The cultures were kept at 37 °C (5% CO_2_), and every third day half of the medium was replaced with fresh medium. After astrocytes became confluent, the culture flasks were shaken for 15–18 h (37 °C, 250 rpm) to remove overlaying microglia and oligodendrocyte precursor cells from the astrocyte layer, the supernatant was discarded, and the astrocytes were passed into new flasks. The astrocytes were ready for use in experiments 12–14 days after the split.

### 4.2. MMSC

Human bone marrow MMSC were obtained from the Research Center of Obstetrics, Gynecology and Perinatology. Their use was approved by the Board of Research Ethics. The research was carried out according to the World Medical Association Declaration of Helsinki, and informed consent was obtained from all subjects. The cells were cultivated in DMEM/F12 (1:1) containing 10% FBS. MMSC were immunophenotyped for CD73, CD90, CD105, as positive markers, and CD14, CD20, CD45, CD34, as negative ones. Ultimately, the cells demonstrated the conventional MMSC phenotype (Suppl. 1).

### 4.3. Co-Cultivation of Rat Astrocytes and MMSC

MMSC used for co-culture experiments were detached and dissociated with 0.05% Trypsin-EDTA, and the suspension was added to cultured adhesive neural cells. The co-cultures were incubated for 24 h in NBM supplemented with 2% FBS for different time intervals.

### 4.4. Oxygen-Glucose Deprivation Protocol

For modeling of oxidative stress caused by OGD [54], the cultured cells were washed twice with Hank’s balanced salt solution (pH 7.3) without glucose and then incubated in this solution in anoxia (using a humidified chamber filled with nitrogen) at 37 °C for 5 h. For control cultures, the culturing conditions remained unchanged throughout the experiment. Immediately after OGD, the saline was replaced with the culture medium.

### 4.5. PC12-ρ0 Cell Line

PC12-*ρ0* cells were generated by the incubation of PC12 cells with 100 ng/mL of ethidium bromide (as described in [55]) for 3 weeks. The *ρ0* cell line was maintained with additional supplementation of 50 μg/mL of uridine and 110 μg/mL of pyruvate.

### 4.6. Confocal Microscopy

The cell cultures were imaged using a LSM510 laser scanning confocal microscope (Carl Zeiss, Oberkochen, Germany). Fluorescence analysis was performed for cells attached to glass-bottom dishes with excitation at 488 nm and 543 nm and emission collected at 500–530 nm and >560 nm, respectively.

### 4.7. Proliferation and Bioenergetics Assays

For the analysis of proliferation, the cells were seeded onto an E-Plate L8 within an iCELLigence system (ACEA Biosciences, San Diego, CA, USA), which had integrated microelectrode sensors in the bottom of the wells, and were incubated at 37 °C with 5% CO_2_ for 150 h. In an iCELLigence system, as the cells proliferate, they adhere to the micro-electrodes, and alterations in electrical impedance reflect the biological status of the cells; therefore, the system allows for the monitoring of time-dependent changes of this parameter. Cell status is expressed as a normalized cell index; cell doubling rate was estimated.

Because of the non-functionality of their mitochondria, *ρ0* cells produced large amounts of lactic acid by anaerobic glycolysis. Lactate levels in the culture medium were determined using a biochemical analyzer A25 (BioSystems, Barcelona, Spain) with an assay in which lactate was oxidized to pyruvate and hydrogen peroxide, which reacts with TOOS to form a colored compound. Lactate concentration was normalized for cell number.

### 4.8. Viral Constructs

MMSC or astrocytes were transfected with 10^5^ TU/mL lentiviral particles encoding mitoGFP or mitoDsRed (Evrogen, Moscow, Russia) and incubated for 3 days, followed by a triple wash with a proper medium; after 24 h, the cells were ready for use for co-culturing. Miro1 overexpression was induced by transfection of MMSC with 10^5^ TU/mL lentiviral particles (produced by Evrogen, Moscow, Russia) encoding Miro1; the Miro1 gene was cloned from RHOT1 Human cDNA ORF Clone (RC214409, OriGene Technologies, Rockville, MD, USA).

### 4.9. Middle Cerebral Artery Occlusion Model of Focal Ischemia

The experimental procedures were conducted in accordance with European Community Council directives 2010/63/EU, and the study was approved by the local institutional animal ethics committee. The experiments were performed on outbred white male rats (320–350 g). The animals had unlimited access to food and water and were kept in cages with a temperature-controlled environment (20 ± 1 °C) with light on from 9 a.m. to 9 p.m. For all surgical procedures, the rats were anesthetized with i/p injection of 300 mg/kg (12%) chloral hydrate. A feedback-controlled heating pad, supplemented with an infrared lamp until the rats awoke, maintained their core temperature (37.0 ± 0.5 °C) during ischemia. Middle cerebral artery occlusion (MCAO) surgery or a sham operation were performed as previously described [56]. Briefly, the right common carotid artery was exposed through a midline cervical incision, and a heparinized intraluminal silicon-coated monofilament with a diameter of 0.25 mm was introduced via the external carotid artery into the internal carotid artery to occlude the blood supply to the middle cerebral artery territory. After 60 min of occlusion, the filament was gently pulled out, and the external carotid artery was permanently closed by cauterization. In sham-operated rats, the right common carotid artery was exposed, and the external carotid artery was electro-coagulated without introducing the filament into the internal carotid artery.

The rats were injected i/v with a suspension of MMSC (3 × 10^6^ cells/kg) and randomly divided into the following groups: (1) Sham + saline (*n* = 6), (2) MCAO + saline (*n* = 8), (3) MCAO + MMSC (*n* = 6), (4) MCAO + MMSC-Miro1 (*n* = 9). The infarct volume was evaluated by analyzing brain T2 weighted MR-images obtained 14 days after the MCAO, as described previously [57]. The ischemic infarct volume for each group was normalized to the mean for the group MCAO + saline. The neurological deficit was estimated by the limb-placing test, consisting of seven tasks, to assess forelimb and hindlimb responses to tactile and proprioceptive stimulation [58]. The rats were habituated to handling and tested before the operation and at the 1st, 3rd, 7th, and 14th post-ischemic days. For each task, the following scores were used: 2 points, normal response; 1 point, delayed and/or incomplete response; 0 points, no response. The total score over seven tasks was evaluated.

### 4.10. Statistics

Statistical analyses were performed using STATISTICA 7.0 for Windows (StatSoft, Inc., Palo Alto, CA, USA). Values are given as mean ± standard error of the mean (SEM). Variance homogeneity was assessed with Levene’s test. Statistical differences in infarct volume between the groups were analyzed using one-way ANOVA with Tukey’s post hoc test. Statistical differences in the limb-placing test scores between the groups were analyzed using the Kruskal–Wallis test with the Mann–Whitney u-test (the Bonferroni post hoc correction was applied). Differences were considered significant at *p* ≤ 0.05.

## Supplementary Materials

The following are available online, Figure S1: Flow cytometry analysis for MMSC phenotyping. The cells were cultivated for three passages, then washed with PBS and incubated with fluorochrome-conjugated antibodies. The levels of СD90, CD105, CD73 were higher than 95%, whereas those of CD14, CD20, CD34, and CD45 were less than 10%.
