# Supplementary figures and images for: Miro1 Enhances Mitochondria Transfer from Multipotent Mesenchymal Stem Cells (MMSC) to Neural Cells and Improves the Efficacy of Cell Recovery
Source: Molecules. 2018 Mar 19;23(3):687. doi: 10.3390/molecules23030687 (PMC6017474; doi:10.3390/molecules23030687)

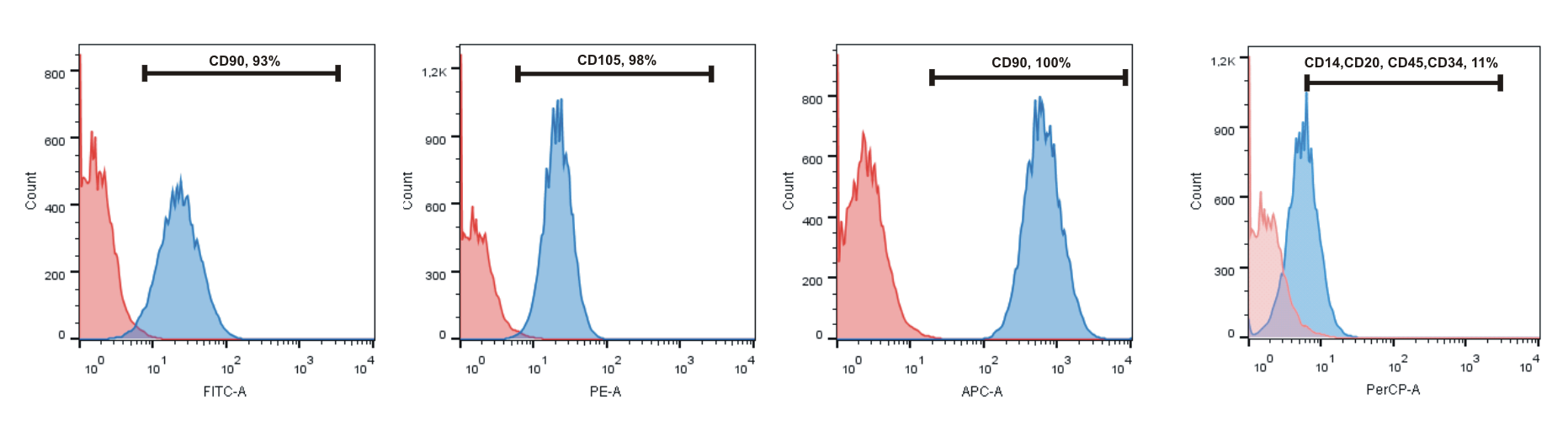

Supplement: Supplementary file 1 [file molecules-23-00687-s001.tif]
